# BOLD fMRI signal characteristics of S1- and S2-SSFP at 7 Tesla

**DOI:** 10.3389/fnins.2014.00049

**Published:** 2014-03-13

**Authors:** Pål E. Goa, Peter J. Koopmans, Benedikt A. Poser, Markus Barth, David G. Norris

**Affiliations:** ^1^MI-Lab, Department of Medical Imaging, St. Olavs University HospitalTrondheim, Norway; ^2^Erwin L. Hahn Institute of Magnetic Resonance Imaging, University Duisburg-EssenEssen, Germany; ^3^Centre for Cognitive Neuroimaging, Donders Institute for Brain, Cognition and Behaviour, Radboud University NijmegenNijmegen, Netherlands; ^4^MIRA Institute for Biomedical Technology and Technical Medicine, University of TwenteEnschede, Netherlands

**Keywords:** 7T, BOLD, fMRI, steady-state free precession, visual cortex

## Abstract

**Object:** To compare the BOLD fMRI signal characteristics at in the cortex and on the pial surface for a non-balanced steady-state free precession sequence (nb-SSFP) at 7 T.

**Materials and Methods:** A multi-echo nb-SSFP sequence was used for high resolution fMRI at 7 T. Two S1 (S^+^) echoes at different echo times were acquired together with an S2 (S^−^) echo. The primary visual cortex (V1) was examined using a reversing checkerboard paradigm at an isotropic resolution of 0.75 mm, with 35 volumes acquired and a total scan time of 27 min.

**Results:** Significant activation was observed in all subjects for all three acquired echoes. For the S1 signal at the longer TE, the activation induced signal change was about 4% in the cortex and 10% at the cortical surface, while for S2 the corresponding values were 3 and 5%.

**Conclusion:** For both S1 and S2 data, the BOLD signal peaks at the pial surface. The large pial surface signal change in S2 may be caused by dynamic averaging around post-capillary vessels embedded within CSF. This is made possible by the long diffusion times of the pathways contributing to the S2 signal and the relatively high diffusion coefficient of CSF. The results indicate that S2-SSFP might not be a suited alternative to spin-echo for high-resolution fMRI at 7 T.

## Introduction

At high magnetic field strengths such as 7 T, T2-weighted sequences are attractive for use in blood oxygenation level-dependent (BOLD) functional MRI (Duong et al., 2002; Yacoub et al., 2003; Olman et al., 2010; Barth and Poser, 2011; Norris, 2012) for two reasons. On the one hand extravascular static dephasing around large draining veins is refocused and does not contribute to the T2-weighted signal, while on the other hand the intravascular signal is small as a result of the short T2 of venous blood at 7 T (Lee et al., 1999; Duong et al., 2003; Yacoub et al., 2005). The dominant contribution to the BOLD contrast in T2-weighted fMRI at 7 T is therefore, expected to be the extravascular dynamic averaging around small vessels, resulting in a BOLD signal mainly from the parenchyma.

The use of spin-echo (SE) based sequences at high field has two drawbacks compared to gradient-echo (GE): the much higher radio frequency (rf) power deposition that often limits the achievable volume coverage and/or requires longer repetition times (TR); and the sensitivity to B1 inhomogeneities. Slice multiplexing (Larkman et al., 2001; Feinberg et al., 2010; Moeller et al., 2010) combined with PINS rf pulses (Norris et al., 2011) offers a potential solution to the SAR problem, while parallel transmit technology (Setsompop et al., 2008) would provide better B1 homogeneity. Non-balanced steady-state free precession (nb-SSFP) has been proposed as an alternative sequence for T2-weighted BOLD fMRI at high field strength (Barth et al., 2010). Nb-SSFP is interesting because of its low rf power deposition and negligible image distortion. It furthermore offers the possibility of simultaneously acquiring the S2 signal together with the S1 signal which respectively resemble the SE and GE signal characteristics. However, an important downside with the nb-SSFP sequence is the low temporal resolution, if only one GE is acquired per TR-interval.

The signal contrast in nb-SSFP differs both from conventional GE and balanced SSFP (Miller et al., 2006). The two different signal types acquired, S1 and S2, have significantly different contrast behavior. The S2 signal, which appears just before each rf pulse, is composed of a sum of SE and stimulated-echo pathways formed by previous excitation pulses. The youngest pathway in S2 is a SE with TE equal to twice the TR of the sequence. The S1 signal appears immediately after the excitation pulse and is composed of a freshly excited FID together with older pathways which it shares with the S2 signal. Hence an S2 image has a SE like contrast, while an S1 image, usually dominated by the fresh FID, has a GE like contrast.

The purpose of this study is to explore the potential of nb-SSFP for high-resolution BOLD fMRI at 7 T. An important motivation for this work comes from our previous study using 3D S2-SSFP which was performed at a coarser spatial resolution (Barth et al., 2010), and showed a high level of similarity between S2-SSFP activation patterns and those recorded for SE EPI. In the absence of a gold standard for brain activation studies a high resolution study should shed some light on the origin of the signal changes, their underlying contrast mechanisms, and potential future uses of nb-SSFP. Specifically, fMRI of the primary visual cortex (V1) at 0.75 mm isotropic resolution is used to compare the signal characteristics of S1 and S2 inside the cortex and at the pial surface, extracted from the simultaneously acquired S1 echoes at two different TE values and the S2 signal. The techniques used herein are too slow for use in fMRI studies: if it were desired to use the S2 signal then the current GE readout would probably best be replaced by a “spiral-in” acquisition. This study does however give insight into both the relative sensitivities of the S1 and S2 signals as well as the contrast mechanisms underlying S1 and S2 activation induced signal changes.

## Materials and methods

### MRI sequence and fMRI acquisitions

**Figure 1:**
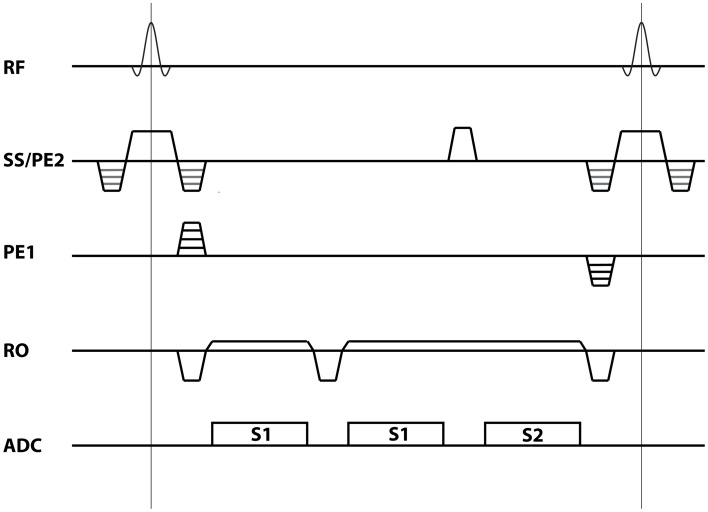
**Sequence diagram.** The multi-echo non-balanced SSFP for the case of two S1 echoes followed by a single S2 echo as used in the current study.

A 3D multi-echo non-balanced SSFP sequence was implemented on a Siemens Magnetom 7 T whole-body scanner (Siemens Healthcare, Erlangen, Germany). The pulse sequence diagram is shown in Figure 1. The sequence allows acquisition of one or more S1-echoes followed by a single S2-echo within each TR. A spoiler gradient was applied between the S1 and S2 echoes to ensure separation of the signals. To boost sensitivity, a custom-built 7-channel surface receive coil array (Orzada et al., 2010) covering the occipital lobe was inserted into the vendor provided 8-channel T/R headcoil (Rapid Biomedical, Rimpar, Germany). Both coils were used for signal reception. Eight subjects were scanned after informed consent was given according to the guidelines of the local ethics committee.

After acquisition of a 3-axis localizer, B0-shimming was performed using a 3D field map calculated from a GRE double echo sequence. A B1-mapping sequence was used to calculate the correct reference rf amplitude in a chosen volume of interest of 5 × 5 × 5 cm centered in the occipital lobe. A sagittal HASTE scan was acquired to accurately identify the calcarine sulcus as a localizer for V1.

The functional 3D acquisition slab consisted of 24 slices with a slice thickness 0.75 mm, axially oriented and tilted toward the coronal plane to align the slab with the calcarine sulcus. In-plane matrix size was 256 × 256 voxels permitting an isotropic voxel size of 0.75 mm. Other sequence parameters were: *FA* = 25°, *BW* = 160 Hz/pix, left-right phase-encoding, inplane GRAPPA acceleration factor 4, *TR* = 27.6 ms, *TE*1 = 7 ms (S1), *TE*2 = 17 ms (S1) and *TE*3 = 23.6 ms (S2). Volume *TR* = 47 s, number of volumes = 35, of which 18 in the reference (“OFF”) condition (black screen, fixation point) interleaved with 17 volumes where a black/white checkerboard reversing at 7.5 Hz was shown (“ON” condition). The total resulting functional scan time was 27 min.

A total of eight subjects were examined, for seven of these the functional scan was followed by an anatomical reference scan using an MP2RAGE sequence (Marques et al., 2010) which had identical orientation, slice thickness and in-plane resolution as the functional scan, but 80 slices to facilitate analysis in FreeSurfer (Dale et al., 1999; Fischl et al., 1999). Parameters were: *TI*1 = 1000 ms, *TI*2 = 3300 ms, *TR* = 8250 ms, echo spacing = 7 ms, *FA* = 4°, *BW* = 190 Hz/pix. For one subject, the anatomical reference scans were obtained on a 3T system (TIM TRIO, Siemens Healthcare, Erlangen, Germany) using the product 32-channel head receive coil and an MPRAGE sequence with parameters *TI* = 1100 ms, *TR* = 2300 ms, *FA* = 10° and *BW* = 130 Hz/px.

For identification of the primary visual cortex V1, retinotopic mapping was performed with a conventional 2D GE-EPI sequence on six subjects at 7T, using a rotating 22.5° dual-wedge (one per hemifield) stimulus of reversing red–green checkerboard (Engel et al., 1997). This dual-wedge rotated clockwise advancing in steps of 22.5° every 5 volumes. The same volume was scanned with the GE-EPI sequence as was acquired during the MP2RAGE using a 2D GE-EPI sequence (20 slices, 64 × 64 matrix, 3 mm isotropic resolution). Other parameters were: *TE* = 25 ms, *FA* = 50°, volume *TR* = 1000 ms, 505 volumes. For two of the subjects, retinotopy scans had previously been obtained at 3 T with similar acquisition parameters.

### Post processing

Initial postprocessing steps for the nb-SSFP data included phase correction using the navigator method described by Goa et al. (2013), realignment of functional series and coregistration with the structural T1-weighted reference volume using SPM8 (Wellcome Centre for Neuroimaging, London, UK). Despite the high spatial resolution no subjects had to be excluded from the analysis due to excessive motion during the scan. The motion parameters were (population mean ± SD): absolute translation = 0.24 ± 0.15 mm, absolute rotation = 0.21 ± 0.11°. Because of the long volume TR, we also estimated the motion during the acquisition of a single volume by calculating the differential motion parameters between each volume and taking the population average. This average “intra-TR” motion was: 0.07 ± 0.02 mm translation and 0.07 ± 0.03° rotation. For visualization of activation maps, voxelwise *t*-scores were calculated and thresholded at *t* = 2.3 after applying an isotropic Gaussian smoothing filter (*SD* = 0.65 voxels) for mild denoising.

### Cortical depth bins

For detailed analysis of the signal at different cortical depths, the structural data were segmented using Freesurfer (Dale et al., 1999; Fischl et al., 1999). The tissue classification was used to extract profiles perpendicular to the cortical surface from white matter, through gray matter and to the pial surface, using the procedure described by Koopmans et al. (2011). Voxels experiencing significant changes in blood flow between the “OFF” and “ON” conditions were identified on the basis of the activation induced phase-changes in the first S1 echo and removed from further analysis. For one subject the phase images were not suitable for blood flow masking and the profile data were excluded from further analysis. Residual small-scale coregistration errors between the structural and functional scans for each subject were taken into account by realigning the sampled through-cortical profiles with each other. For each profile the position of the maximum gradient of the S2 signal (corresponding to the gray matter—CSF boundary) was detected and for all profiles these points were aligned. Only profiles were included for which such a gradient peak was detected less than 10% away from the original position as defined by the Freesurfer segmentation. This differs from the procedure in (Koopmans et al., 2011) where two edges in the functional profiles were detectable and both alignment and scaling were performed. Cortical profiles (*N* = 1082 ± 506, mean ± SD) were successfully extracted from seven subjects. Since the anatomical information available in the nb-SSFP images allowed reliable detection of only the CSF-GM boundary on individual profiles, we could not establish sufficient accuracy of the profile alignment to analyse the data on a full layer-specific level. High quality profiles for laminar analysis that we have previously demonstrated (Koopmans et al., 2010, 2011) rely on the accurate detection of both the CSF-GM boundary and the GM-WM boundary in order to realign and rescale the profiles. The residual spatial broadening of profiles in the current data is expected to increase gradually from the CSF-GM boundary toward the GM-WM boundary. A worst-case estimate of the width of this broadening can be found from the variation in cortical thickness within V1. From our own freesurfer data this was estimated to around 0.6 mm or 30% of the average V1 cortical thickness. Hence all further analysis on the data was performed after classifying the profiles into four bins; deep GM, middle GM, upper GM, and pial surface.

### Signal changes

The signal changes for each echo and subject were calculated using the signal intensity averaged across the activated state “ON” and resting state “OFF” volumes, both in absolute terms:

(1) $$\Delta S={\bar{S}}_{On}-{\bar{S}}_{Off}$$

and in percentage of the “OFF” condition signal in each bin:

(2) $$\Delta S=100^{*}\left[{\bar{S}}_{On}-{\bar{S}}_{Off}\right]/{\bar{S}}_{Off}.$$

This is done to illustrate the differences between these two ways of stating the BOLD signal amplitude. In simulations, the relative (or percentage) BOLD signal is usually used, while in the context of comparing (and possibly summing) the BOLD signal amplitude from different tissues and compartments *in vivo*, the absolute signal change in each compartment is more relevant. Also, depending on the noise characteristics of the image data, the relative or absolute signal change correlates best with the statistical value for the activation.

## Results

**Figure 2:**
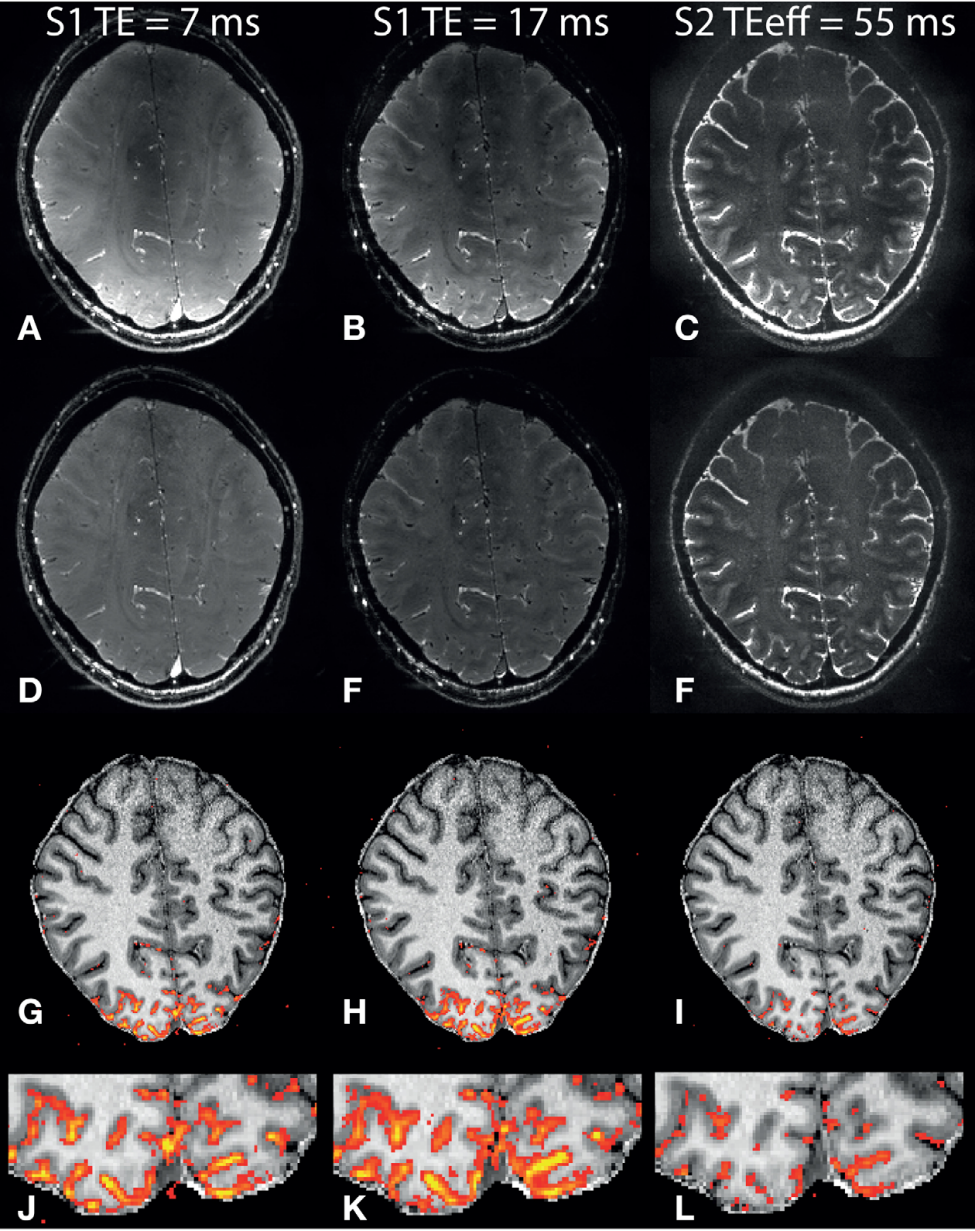
**Example nb-SSFP images and activation maps.** Mean resting state images from one subject: **(A)** S1, *TE* = 7 ms, **(B)** S1, *TE* = 17 ms, **(C)** S2. **(D–F)**: Same as **(A–C)** after bias field correction. **(G–I)**: Anatomical image (MP2RAGE) overlaid with thresholded *t*-score maps for the first S1 **(G)**, second S1 **(H)** and S2 **(I)** echoes. **(J–L)**: Zoomed view of the same thresholded *t*-score maps. Color range for *t*-values is 2.3–10, and *t*-value images were smoothed using a gaussian kernel with *SD* = 0.65 voxels.

Nb-SSFP images (mean over “OFF” condition) from one subject are shown in Figures 2A–C. There is a strong reduction in signal intensity in the sagittal sinus when going from the first S1 echo at 7 ms (Figure 2A) to the second S1 echo at 17 ms (Figure 2B). This illustrates the short T2^*^ of venous blood at 7 T. Similarly in the S2 echo (Figure 2C), for which the shortest coherence pathway has *TE* = 55.2 ms, no signal from the sagittal sinus can be observed, while CSF with its long T2 is still bright. For better visibility, the intensity in the S2 image has been scaled up by a factor of 3 relative to the two S1 echoes due to the much lower S2 signal intensity. Bias field corrected images are shown in Figures 2D–F, which compensate for the effects of B1-transmission inhomogeneity at the center of the brain, and the fall-off in receiver sensitivity with distance from the occipital coil.

Smoothed and thresholded *t*-score maps (*t* = 2.3) are shown overlaid on the coregistered T1-weighted images in Figures 2G–L. Significantly activated voxels are observed for all three echoes, and with a similar activation pattern. In general the *t*-values are higher for S1 compared to S2, and the highest *t*-values are observed in the long –TE S1 echo.

**Figure 3:**
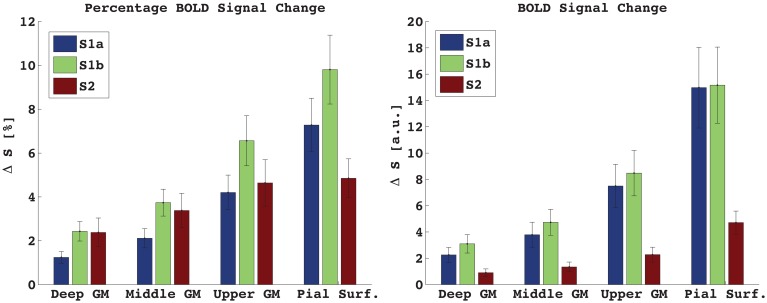
**Percentage (left) and absolute (right) signal change in GM and at the pial surface.** S1a, *TE* = 7 ms, S1b, *TE* = 17 ms. Mean ± s.e.m., *n* = 7. Due to low GM-CSF contrast in S1, the absolute and percentage signal change looks very similar. In S2, where CSF is much brighter than GM, the percentage signal change is less different between bins than for the absolute signal change.

**Figure 4:**
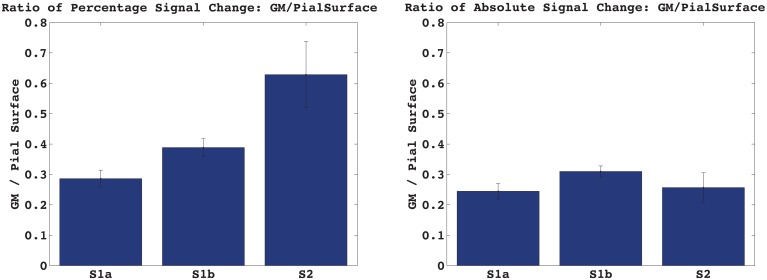
**Activation induced signal change in GM vs. at the Pial surface.** The ratio of the absolute signal change in the cortex vs. at the pial surface is very similar for S1 and S2, contrary to what is expected from a T2-weighted vs. a T2^*^-weighted signal.

Figure 3 shows the percentage and absolute signal changes in each of the four bins for all three acquired echoes. For both S1 and S2 the signal change increases monotonically along the profile and peaks at the pial surface. The S1 signal change is 3.5–4% in the center of gray matter, and around 10% at the pial surface, as measured against the local baseline intensity. This corresponds to a cortex-to-surface BOLD signal ratio of 0.4, see Figure 4. For S2, the cortical signal change was also in the range of 3.5%, while the pial surface signal was around 5%. This corresponds to a cortex-to-surface BOLD signal ratio of 0.65. In absolute terms however, the S2 cortex-to-surface BOLD signal ratio was only about 0.25. This is due to the hyper-intense CSF compared to GM in S2.

## Discussion

### Activation induced signal change in S1 echoes

The S1 echo is dominated by the fresh FID and is therefore GE-like and can be considered T2^*^ weighted, however it also depends indirectly on T2 and is hence not identical to a pure GE signal. Tissue with short T2 will tend also to have shorter T1 values: the short T2 will tend to reduce SSFP signal intensity, wheras a short T1 will increase it, giving rise to the well known blandness of the S1 signal contrast. Hence any direct comparison between the observed signal change in this study and studies using GE-sequences must be performed with caution.

The vascular density varies across the cortex (Duvernoy et al., 1981), where cortical layer IV (part of vascular layer 3), which is particularly thick in human V1, has the highest vascular density. It is expected that this variation in vascular density will modulate the BOLD signal across the cortex. Our group (Koopmans et al., 2010) observed a peak in the signal change localized to the middle vascular layer in gray matter, using a conventional GE sequence at 0.75 mm isotropic resolution at 3T. In multi-echo experiments performed at 7T we showed that the profile will vary significantly with TE, owing to the variation in T2^*^ between layers (Koopmans et al., 2011). With this insight it is possible to explain why Polimeni et al. (2010), using a 2D GE-EPI at 1 mm isotropic resolution at 7T did not observe any central peak at a TE of 24 ms; and Harel et al. (2006) observed a small central peak in the GE signal change in cat visual cortex at 9.4 T using a TE of 20 ms. In the current study we find a monotonically increasing BOLD signal across the three bins defined in gray matter, resembling the results shown in Figure 6 in Polimeni et al. (2010). Based on the varying reports on the detection of this peak in GE-fMRI, we cannot establish whether the absence of a peak in our data suggests a different signal characteristics in S1-SSFP compared to GE, or it is simply caused by insufficient spatial specificity as a result of it only being possible to define the gray-matter CSF boundary but not the gray-matter white matter boundary in the current functional data. The point spread function of the nb-SSFP sequence could potentially affect the spatial resolution of the functional imaging data. Since the volumes were acquired contiguously the nb-SSFP sequence is in a steady state for all k-space components, and hence the point spread function should be unaffected. The total readout duration of 6 ms is short compared to T2^*^ of around 30 ms in gray matter, but will give a variation of maximum 20% in the weighting of different k-space components. This however is in the same order as previous studies (Koopmans et al., 2011) where the central peak was observed, and should be a negligible effect.

### Activation induced signal change in S2 echo

The strong activation signal at the pial surface in S2 is somewhat surprising because T2-weighted fMRI is generally expected to be less sensitive to the contribution from large veins, since the static dephasing surrounding them should no longer contribute to the contrast. However, recent simulations suggest that the contribution from pial vessels embedded in CSF is less suppressed in SE-fMRI at 7 T than previously believed (Pflugfelder et al., 2011). A pial surface peak has previously been observed in SE-fMRI at high field strength. Goense et al (Goense and Logothetis, 2006) found a surface peak in monkey V1 at 4.7 T. There, the amplitude of the peak varied with the duration of the EPI readout, strongly suggesting the surface peak was caused by the T2^*^-weighting induced by the finite EPI-readout duration in SE-EPI and not by T2 changes. At short EPI readouts of 7.7 ms the surface peak disappeared. Harel et al. (2006) observed a surface peak in cat visual cortex at 9.4 T with an EPI readout of 22.4 ms, comparable to the results for the same EPI readout duration in (Goense and Logothetis, 2006). The work by Jin and Kim (2008), in contrast reports a flat SE-EPI signal change through all layers of the cortex, although on the basis of known vascular density profiles (Duvernoy et al., 1981) and SE contrast mechanisms, a peak should be expected at vascular layer 3. In our study the readout duration of each echo is about 6 ms, which is short compared to the EPI readouts used in previous studies. On the other hand the S2 echo is acquired asymmetrically, in the sense that the center of k-space is acquired 4 ms before the center of the refocusing S2 echo which forms at the center of the following RF pulse. In addition, the readout itself was asymmetric. These factors make a direct comparison to the centered EPI-readout used in the above-mentioned studies difficult. However, it is plausible that the pial surface peak is at least partly due to residual T2^*^ contrast caused by both the finite readout duration and its asymmetry.

The S2 SSFP signal differs from a conventional SE sequence in terms of its summation of different signals. In particular, not only the “youngest” or shortest SE contributes to the signal, but older and longer pathways of both spin echoes and stimulated echoes exist with additional time for diffusion to occur. In a recent paper (Goa et al., 2013) we estimate the effective lifetime of the S2 signal to about 0.3 s for relaxation times and sequence parameters relevant to the ones used in the current study. In addition, the diffusion coefficient in CSF is a factor 3–4 larger than in gray matter. Put together, this results in an effective diffusion length for the S2 signal in CSF of about 70 μm. The diameter of the collecting veins toward and at the pial surface range from 20 to 200 μm (Duvernoy et al., 1981), so an extravascular contribution from dynamic averaging around post-capillary vessels is very likely, in particular for the “oldest” parts of the S2 SSFP signal.

### Stability of the S2 signal

The S2 signal is sensitive to the effects of flow and motion because numerous coherence pathways contribute to the signal, and the first moments of the read and slice gradients are non-zero. This has the consequence, that moving to a higher spatial resolution, as was done in this study, will increase this sensitivity. As we recorded both S1 and S2 signals in the current study it was not possible here to desensitize the S2-signal to motion. In an experimental design in which only the S2 signal should be measured this would be possible using gradient moment nulling (Wood et al., 1991), if an EPI or spiral readout were to be used then this could in theory also be motion nulled. In the current paper there was a very low level of subject motion, and the signal from larger vessels was excluded from the analysis. Hence the general conclusions of this paper are largely unaffected by motion, however this could be an important consideration were S2 signals to be considered for routine use in fMRI.

## Conclusion

We have demonstrated the capability of multi-echo nb-SSFP for high-resolution BOLD-fMRI at 7 T, with simultaneous acquisition of S1 and S2 signals. Activation induced signal changes were observed in both the S1 and the S2 signals. The activation results suggest that the functional contrast of S1 and S2 nb-SSFP are not equivalent to GE and SE, respectively. In particular, the ratio between the BOLD signal originating from within the cortex vs. on the cortical surface, are almost the same for S1 and S2. This indicates that S2-SSFP might not be a suited alternative to SE for fMRI at 7 T. A non-equivalence between the S2 signal and that of SE EPI was recently reported for 9.4 T in humans (Ehses et al., 2013). We attribute the large S2 BOLD signal at the cortical surface to dynamic averaging around post capillary vessels within the CSF due to long diffusion times for the S2 signal.

### Conflict of interest statement

The authors declare that the research was conducted in the absence of any commercial or financial relationships that could be construed as a potential conflict of interest.
